# Core Outcome Measures in Effectiveness Trials (COMET) initiative: protocol for an international Delphi study to achieve consensus on how to select outcome measurement instruments for outcomes included in a ‘core outcome set’

**DOI:** 10.1186/1745-6215-15-247

**Published:** 2014-06-25

**Authors:** Cecilia A C Prinsen, Sunita Vohra, Michael R Rose, Susanne King-Jones, Sana Ishaque, Zafira Bhaloo, Denise Adams, Caroline B Terwee

**Affiliations:** 1 Department of Epidemiology and Biostatistics, VU University Medical Center, Van der Boechorststraat 7, 1081 BT Amsterdam, The Netherlands; 2 EMGO Institute for Health and Care Research, VU University Medical Center, Van der Boechorststraat 7, 1081 BT Amsterdam, The Netherlands; 3 Department of Pediatrics, Faculty of Medicine and Dentistry, and School of Public Health, University of Alberta, Edmonton Continuing Care Center 8B19, 11111 Jasper Avenue, Edmonton, AB T5K 0L4, Canada; 4 Women’s and Children’s Health Research Institute, University of Alberta, 4-081 Edmonton Clinic Health Academy (ECHA), 11405 - 87 Avenue, Edmonton, AB T6G 1C9, Canada; 5 Department of Neurology, King’s College Hospital, Denmark Hill, London SE5 9RS, UK

**Keywords:** COMET, Core outcome set, Delphi study, Outcome measurement instruments, Selection, Protocol, Guideline

## Abstract

**Background:**

The Core Outcome Measures in Effectiveness Trials (COMET) initiative aims to facilitate the development and application of ‘core outcome sets’ (COS). A COS is an agreed minimum set of outcomes that should be measured and reported in all clinical trials of a specific disease or trial population. The overall aim of the Core Outcome Measurement Instrument Selection (COMIS) project is to develop a guideline on how to select outcome measurement instruments for outcomes included in a COS. As part of this project, we describe our current efforts to achieve a consensus on the methods for selecting outcome measurement instruments for outcomes to be included in a COS.

**Methods/Design:**

A Delphi study is being performed by a panel of international experts representing diverse stakeholders with the intention that this will result in a guideline for outcome measurement instrument selection. Informed by a literature review, a Delphi questionnaire was developed to identify potentially relevant tasks on instrument selection. The Delphi study takes place in a series of rounds. In the first round, panelists were asked to rate the importance of different tasks in the selection of outcome measurement instruments. They were encouraged to justify their choices and to add other relevant tasks. Consensus was reached if at least 70% of the panelists considered a task ‘highly recommended’ or ‘desirable’ and if no opposing arguments were provided. These tasks will be included in the guideline. Tasks that at least 50% of the panelists considered ‘not relevant’ will be excluded from the guideline. Tasks that were indeterminate will be taken to the second round. All responses of the first round are currently being aggregated and will be fed back to panelists in the second round. A third round will only be performed if the results of the second round require it.

**Discussion:**

Since the Delphi method allows a large group of international experts to participate, we consider it to be the preferred consensus-based method for our study. Based upon this consultation process, a guideline will be developed on instrument selection for outcomes to be included in a COS.

## Background

There is a lack of consensus with regard to the selection of outcomes and outcome measurement instruments for clinical trials. This has resulted in different outcomes being measured and a variety of instruments being used for measuring the same outcome. This may cause inconsistencies in the outcomes reported and difficulties in comparing these outcomes in systematic reviews and meta-analyses [1]. In addition, there is great variability in the quality (for example, in reliability and validity) of outcome measurement instruments used and it is not always clear if the best instrument is being used for a given outcome. To overcome these issues, standardisation of the selection of outcomes and outcome measurement instruments is needed.

The Core Outcome Measures in Effectiveness Trials (COMET) initiative, launched in January 2010, aims to facilitate the development and application of agreed standardized sets of outcomes, also known as ‘core outcome sets’ (COS). A COS is an agreed minimum set of outcomes that should be measured and reported in all clinical trials of a specific disease or trial population. Although a COS is disease or population specific, it is not trial specific; it is a recommendation of *what* should be measured and reported in all clinical trials [2]. Initiatives such as Outcome Measures in Rheumatology (OMERACT), Harmonizing Outcome Measures for Eczema (HOME), and TREAT-NMD Neuromuscular Network currently work on the development and application of agreed standardized sets of outcomes for rheumatic diseases, atopic dermatitis, and neuromuscular disease respectively. Once COS are defined, it is then important to achieve consensus on *how* these outcomes should be measured.

The Core Outcome Measurement Instrument Selection (COMIS) project is a joint initiative between COMET and COnsensus-based Standards for the selection of health Measurement INstruments (COSMIN). The COSMIN initiative aims to improve the selection of outcome measurement instruments and, in pursuit of this aim, it has developed standards for assessing the methodological quality of studies exploring the measurement properties of outcome measurement instruments [3]. In addition, COSMIN has developed search filters and a protocol for performing systematic reviews of outcome measurement instruments [4]. The COMIS project aims to build upon both the COMET and COSMIN initiatives with the ultimate aim of developing a guideline on how to select outcome measurement instruments for outcomes to be included in a COS. It is expected that such a guideline will support COS developers in the task of selecting outcome measurement instruments, thereby meeting the agreed standards for each of the outcomes included in a COS. When selecting outcome measurement instruments, a number of aspects need to be considered *a priori*, such as the construct(s) to be measured, the target population, and the goals of treatment. In addition to this, a number of tasks need to be performed, for example a literature search to find potentially relevant outcome measurement instruments for a particular outcome, and a quality assessment of the available instruments.

Currently, no guidelines are available to support outcome measurement instrument selection. The aim of the present study is to achieve consensus on the methods for selecting outcome measurement instruments for outcomes to be included in a COS. If there is an absence of empirical evidence to inform such a consensus then different methods can be used to synthesize the opinions of experts based on their expertise and scientific background. These methods are increasingly being used to develop guidelines that are used in healthcare [5]. Three main approaches exist: the Delphi method, the nominal group technique (also known as expert panel), and the consensus development conference [6]. All three methods involve reaching consensus. However, in a Delphi study the group does not need to meet, which confers anonymity, and opinions are to be expressed free from group pressure. This is our rationale for undertaking a Delphi study. The results of the Delphi study will be used to inform the content of the guideline on how to select outcome measurement instruments for outcomes included in a COS. In addition to the results of the Delphi study, input from other resources will also be used, such as from COSMIN and the Primary Outcomes Reporting in Trials (PORTal) initiative which examines the reporting and validation of outcome measurement instruments in clinical trials [7].

We believe that an agreed guideline on outcome measurement instrument selection will promote and supplement the development and use of COS with the advantage of improving the standard of all clinical trials.

## Methods/Design

### Delphi method

**Figure 1:**
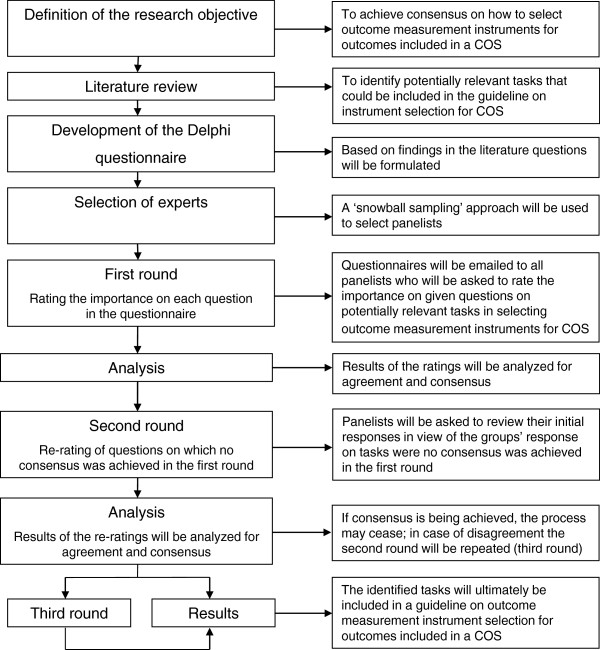
Overview of the Delphi method.

A Delphi study, informed by a literature review, is being performed aiming to achieve consensus on relevant tasks that need to be performed when selecting instruments for outcomes to be included in a COS (Figure 1).

### Literature review

A literature review was performed to identify existing studies that provide relevant information on the development of guidance on outcome measurement instrument selection. This literature review also helped us by identifying relevant stakeholders and international experts that were invited to participate in the Delphi study.

#### Eligibility criteria

The following types of studies were included: guidelines, reviews, systematic reviews, meta-analyses, and protocols that develop or apply methodology for selecting outcomes or outcome measurement instruments to be used in clinical trials. Studies that discuss how to measure rather than how to select outcomes or outcome measurement instruments for use in clinical trials were excluded. Studies that mainly discussed performance characteristics of outcome measurement instruments were excluded as well.

#### Literature search

An electronic literature search was conducted by a research librarian (SKJ) to identify studies that provide relevant information on instrument selection. The search strategy was developed by consulting search strategies outlined by Reeve *et al.* who performed a study on the development of minimum standards for patient-reported outcome measures [8], Terwee *et al.* who developed a methodological PubMed search filter for finding studies on measurement properties of outcome measurement instruments [4], Sinha *et al.* who performed a systematic review of studies that address the selection of outcomes to measure in clinical trials in children [9], Brettle *et al.* who performed a study about searching for information in MEDLINE efficiently and effectively [10], and by search terms gathered from the online MEDLINE thesaurus and medical subject headings (MeSH). The search was limited to English language articles and humans, and there were no restrictions for publication dates. Searches were performed in the following databases: MEDLINE, CINAHL, Embase, and PsycINFO. Our search strategy is presented in full in Table 1.

**Table 1:** Search strategy

	**Search terms**
1.	“Outcome Assessment (Health Care)”/
2.	Clinical Trials as Topic/
3.	guidance.ti,kw.
4.	guideline*.ti,kw.
5.	(outcome* adj2 measure*).ti,kw.
6.	outcome*.ti,kw.
7.	treatment outcome.ti,kw.
8.	outcome studies.ti,kw.
9.	outcomes assessment.ti,kw.
10.	(outcome* adj2 instrument*).ti,kw.
11.	measurement instrument*.ti,kw.
12.	1 or 2
13.	3 or 4 or 5 or 6 or 7 or 8 or 9 or 10 or 11
14.	12 and 13
15.	limit 14 to (guideline or meta analysis or “review”)
16.	limit 15 to English language
17.	limit 16 to humans

#### Selection of articles

An initial selection of articles was performed by a research librarian (SKJ), who excluded references that were not guidelines, reviews, systematic reviews, meta-analyses, or protocols. Two reviewers (SI and ZB) selected articles based on titles and abstracts that were identified from the literature searches, taking the predefined inclusion criteria into account. Differences in selection were discussed and, based on consensus by the two reviewers, a decision was made whether the article fulfilled the eligibility criteria. In case of disagreement between the two reviewers, a third independent reviewer (SV) was consulted.

### Development of the Delphi questionnaire

From the literature review potentially relevant tasks on instrument selection were identified and included in the Delphi questionnaire. These tasks consist of, but are not limited to: (1) conceptual considerations*,* for example a description of the construct(s) to be measured, the measurement aim (a discriminative, predictive, or evaluative purpose), and the target population for which the instrument is intended; (2) methods of finding relevant outcome measurement instruments*,* for example by using existing systematic reviews of instruments on a specific construct or, in the absence of an existing systematic review, by performing a systematic review of instruments, by using existing databases of instruments, or by performing (additional) literature searches; (3) methods of evaluating outcome measurement instruments*,* for example by describing and comparing the qualitative attributes (number of items, response categories, and so forth), content, and measurement properties of the instruments, and considering the feasibility of the application of the instrument; (4) methods for applying quality criteria for good outcome measurement instruments*,* for example minimal criteria for good measurement properties or minimal standards for selecting an instrument; (5) performing validation studies if necessary; and (6) methods for selecting the most appropriate instrument for each outcome included in a COS*.* Questions were formulated on the relevance of each of the tasks to be included in the Delphi questionnaire.

#### Definitions

Similar constructs are defined differently across several research groups such as COMET, OMERACT, and HOME. As there is currently no consensus on the definitions, we would like to explicitly state the definitions that are being used in the COMET Delphi study in order to avoid any possible misinterpretations.

### Core outcome set (COS)

A COS is an agreed minimum set of outcomes that should be measured and reported in all clinical trials of a specific disease or trial population. A COS includes all relevant outcomes of a specific health condition within a specified setting (the OMERACT definition refers to ‘core domain set’ whereas the HOME definition refers to ‘core outcome domains’).

### Outcome

An outcome refers to *what* is being measured, also referred to as a concept, construct, or (sub)domain. In the context of a clinical trial it refers to any identified result in an outcome arising from exposure to a causal factor or a health intervention (the OMERACT definition refers to ‘(sub)domain’ whereas the HOME definition refers to ‘outcome domain’).

### Outcome measurement instrument

An outcome measurement instrument refers to *how* the outcome is being measured (the tool used to assess the outcome). An outcome measurement instrument can be a single question, a questionnaire, a performance-based test, a physical examination, a laboratory measurement, an imaging technique, and so forth (the HOME definition refers to ‘outcome measure’).

### Outcome parameter

An outcome parameter refers to any identified result in an outcome arising from exposure to a causal factor or a health intervention (the OMERACT definition refers to ‘outcome’).

The terms and definitions of measurement properties will be adopted from COSMIN [11].

### Selection of experts

The selection of panelists should reflect the population that is intended to use the guideline for instrument selection. In order to enhance the credibility and acceptance of the guideline, the panelists should be selected from a diverse range of institutions and organizations that may facilitate the dissemination and implementation of the guideline for instrument selection [5].

A ‘snowball sampling’ approach was used to select panelists for the Delphi study. The identification of relevant stakeholders and experts began with a preliminary list of experts (defined for these purposes as people who have a credibility relating to the target audience as indicated by, for example, authorship of multiple frequently cited publications in this field). These experts were selected from the following institutions and/or working groups: Cochrane Patient Reported Outcomes (PRO) Methods Group; COMET initiative, COSMIN Delphi panel and Steering Committee; Equator Network; HOME initiative; Idea, Development, Exploration, Assessment, Long-term follow-up (IDEAL) collaboration; International Spinal Cord Society; International Society for Quality of Life Research (ISOQOL); North West Hub for Trials Methodology Research; OMERACT initiative; PORTal initiative; Standard Protocol Items: Recommendations for Interventional Trials (SPIRIT) group; Standards for Research in Child Health (StaR Child Health) Steering Group; and TREAT NMD alliance. In addition, editors of important relevant journals, such as *Quality of Life Research* and the *Journal of Clinical Epidemiology*, were invited to participate as well. The preliminary list was then augmented with authors who have published in relevant topics, mostly informed by the literature search. Finally, stakeholders and experts were given the opportunity to add whomever else they felt should be included as a relevant stakeholder. To facilitate the dissemination and implementation of the final guideline we intend to be inclusive of relevant stakeholders and/or perspectives. We found no guidelines for the sample sizes of Delphi studies but in general, having more panelists will increase the reliability of group judgment [5]. In the COSMIN Delphi study, 70% of the invited experts agreed to participate [3,12], but as the topic of the present study is much broader we anticipated a lower response rate (between 30 and 40%). We therefore invited all 481 experts identified in the methods outlined above to participate in this Delphi study.

### First round

The Delphi method involves in a series of rounds in order to achieve consensus among a panel of experts [6].

In the first round, a link to the electronic version of the Delphi questionnaire, including instructions for completion, was sent by email to 120 panelists (24.9%) who accepted our invitation to participate. In the first round, panelists were asked to anonymously rate the relevance of different tasks of three main themes on outcome measurement instrument selection. They were asked to rate the importance of conceptual considerations, the relevance of different tasks in finding instruments, and the required evidence and minimal standards for measurement properties of instruments to be included in a COS. Responses could be given on a multiple response scale, for example ‘highly recommended’, ‘desirable’, ‘not relevant’, or ‘not my expertise’. Examples of questions on the conceptual considerations in the selection of outcome measurement instruments include: ‘Should COS developers agree in detail upon the constructs to be measured before starting to search for outcome measurement instruments?’; ‘Should COS developers agree upon the target population before starting to search for outcome measurement instruments?’; and ‘Should COS developers agree for each outcome upon the type of instrument to be used before starting to search for outcome measurement instruments?’. Examples of questions on finding relevant outcome measurement instruments include: ‘Should COS developers search for existing systematic reviews of outcome measurement instruments?’ and ‘Should COS developers search in electronic literature databases (for example PubMed or Embase) to find relevant outcome measurement instruments?’. Examples of questions on the evaluation of outcome measurement instruments include: ‘Should evidence be available on the internal consistency of outcome measurement instruments to be included in a COS?’ and ‘What should be the minimum standard for internal consistency for an outcome measurement instrument to be included in a COS?’.

Panelists were encouraged to justify their choices in free text boxes, which could be supported by relevant literature, and to add other possibly relevant tasks. Subsequently, panelists were asked for their opinion on whether the methods for selecting outcome measurement instruments for a COS are similar to the methods for selecting outcome measurement instruments for individual clinical trials. This question was to determine whether panelists felt that the same guidelines could be applied to an individual clinical trial. Panelists were asked to respond within three weeks. To increase the response rate, an email reminder was sent after two weeks.

A total of 95 panelists (79.2%) completed the first questionnaire, representing 14 countries. Most of the panelists came from the Netherlands (*N* = 19), Australia (*N* = 15), Canada (*N* = 14), and the UK (*N* = 12). Brazil, Norway, Portugal, and Switzerland were represented by one panelist each. Of the panelists, 42.1% were epidemiologists, 31.6% were allied healthcare professionals, 30.5% were clinimetricians or psychometricians, 29.5% were medical doctors, and 10.5% were statisticians. A total of 15.8% indicated that they had another academic background. The current professions of the panelists included researchers (92.6%), clinicians (27.4%), and journal editors (9.5%). Thirty panelists (31.6%) indicated that they had ‘no experience’ in COS development, 28 panelists (29.5%) indicated to have ‘some experience’, 26 (27.4%) had ‘a little experience’, and 11 (11.6%) had ‘a lot of experience’.

### Second round

The responses of this panel are currently being aggregated and will be fed back to all panelists anonymously in a feedback report as part of the second round. In the second round, panelists will be asked to review their initial responses on those tasks not achieving consensus in the first round in the light of the groups’ responses.

### Analysis

We define consensus on a task to have been reached when at least 70% of the panelists considered the task ‘highly recommended’ or ‘desirable’ and if no opposing arguments are provided. Tasks on which such consensus is reached will be included in the guideline and panelists will not be asked to vote for these tasks again. When at least 50% of the panelists consider a task ‘not relevant’ and when no strong arguments in favor of this task are given, we plan to exclude the task from the guideline. Tasks which attract an indeterminate response will be taken to the second round of this Delphi consultation [6,13].

Based on the responses given in the first round, including the comments given in the free text boxes, proposals will be formulated. Panelists will be asked to rate their agreement on the given proposals. Response options will include ‘strongly agree’, ‘agree’, ‘no opinion’, ‘disagree’, and ‘strongly disagree’. Additionally, new questions that may arise based on the comments given, will be formulated. These questions will be marked as ‘new questions’ and panelists will be asked to rate the relevance of these tasks in the second round. The results of the second round will then again be analysed for consensus following the same procedure as for the first round.

### Third round

Tasks from the second round that remains indeterminate will be taken to the third round. In the third round, the results from the second round will again be fed back to all panelists anonymously in a feedback report and panelists will again be asked to re-rate the indeterminate tasks in view of the groups’ response. For tasks that come back as contentious even after round three, the COMIS Steering Committee (CP, CT, MR, and SV) will plan a face-to-face meeting to discuss these tasks. A third round will be omitted if the results of the second round suggest that consensus is being obtained following our predefined consensus criteria.

### Results

Results will be anonymously reported in a consensus report which will be distributed among all panelists who participated in the Delphi study. This report will include an indication of the distribution of panelists’ ratings, including their comments and suggestions. Results will be presented both quantitatively (median scores and the interquartile ranges) and qualitatively (listings of the comments and suggestions given by the panelists). The identified tasks will ultimately be included in a guideline on outcome measurement instrument selection for outcomes to be included in a COS.

### Ethics

As this project does not involve patients or study subjects, according to the Dutch Medical Research in Human Subjects Act (WMO) it is exempt from ethical approval in The Netherlands. No ethical approval is required in the UK for identical reasons. Ethical approval has been obtained from the ethical committee of the University of Alberta, Canada.

## Discussion

When insufficient or contradictory information is available on a certain topic, consensus-based methods such as a Delphi method are generally considered to be appropriate methods to determine the extent to which experts agree on that topic. Since the Delphi method allows a large group of international experts to participate, we consider it to be the preferred method for our study. A Delphi method is relatively less expensive and overcomes some of the disadvantages, or limitations, that are generally found with decision-making processes in groups or committees. Performing an anonymous Delphi study by email may avoid dominance of certain persons in face-to-face group meetings. Secondly, feedback is provided in a controlled manner [14,15]. The results of a Delphi study are highly dependent upon the composition of the panel. Therefore, we aimed to include a sample of experts in the field who represent diverse institutes and organizations and reflect the population that is intended to use a guideline for outcome measurement instrument selection. However, it is difficult to examine the representativeness of the panelists as it is impossible to draw a random sample from all experts. Experts were therefore selected non-systematically which may be considered as a limitation of our study.

The ultimate aim of the COMIS project is to support COS developers on how outcomes included in COS should be defined and measured by providing a guideline on outcome measurement instrument selection. To this aim, eight different tasks are currently being executed, of which the Delphi study is one of them. The specific objectives of these tasks are: (1) to identify existing research and relevant partners in the field of instrument selection; (2) to provide guidance and support to COS developers on how to find all measurement instruments that measure a specific core outcome; (3) to identify the essential information that should be gathered for each measurement instrument identified, including characteristics of the instrument and information on measurement properties; (4) to collect available systematic reviews of measurement instruments; (5) to provide guidance and support to COS developers on how to perform systematic reviews of measurement instruments; (6) to provide guidance and support to COS developers on selecting instruments for COS; (7) to disseminate findings on measurement instruments among COS developers; and (8) to develop a workshop on evidence-based instrument selection for COS developers. The results of each task will be used as input for the final guideline on outcome measurement instrument selection for outcomes to be included in COS. The guideline will promote the development and use of COS, which will ultimately improve the conducting and reporting of clinical trials and enhance the value of evidence synthesis by reducing heterogeneity between trials.

## Trial status

The Delphi study is currently ongoing. It is anticipated that the second Delphi questionnaire will be sent to all panelists who agreed to participate in the second round in May 2014. A published guideline on the methods for selecting outcome measurement instruments to be included in a COS is expected by the end of 2014.

## Abbreviations

COMET: Core Outcome Measures in Effectiveness Trials; COMIS: Core Outcome Measurement Instrument Selection; COS: Core Outcome Set; COSMIN: COnsensus-based Standards for the selection of health Measurement INstruments; HOME: Harmonising Outcome Measures for Eczema; IDEAL: Idea, Development, Exploration, Assessment, Long-term follow-up; ISOQOL: International Society for Quality of Life Research; NMD: Neuro Muscular Diseases; OMERACT: Outcome Measures in Rheumatology; PORTal: Primary Outcomes Reporting in Trials; PRO: Patient Reported Outcome; SPIRIT: Standard Protocol Items: Recommendations for Interventional Trials; StaR Child Health: Standards for Research in Child Health.

## Competing interests

The authors declare that they have no competing interests.

## Authors’ contributions

CT is the principle investigator. CP is the coordinating investigator. CT and CP are responsible for the design of the study and the study protocol. SKJ is responsible for the search of the literature review. SV, MR, SKJ, SI, ZB, and DA have contributed to the design of the study and/or to the content of the study protocol with important intellectual revisions. CP is responsible for drafting the protocol manuscript. All authors have read and approved the final protocol manuscript.
